# Experiments in modeling recent Indian fertility pattern

**DOI:** 10.1038/s41598-021-85959-z

**Published:** 2021-03-23

**Authors:** Ujjaval Srivastava, Kaushalendra Kumar Singh, Anjali Pandey, Neeraj Narayan

**Affiliations:** 1 Department of Statistics, Banaras Hindu University, Varanasi, India; 2 Ministry of Tourism, Government of India, New Delhi, India; 3 Department of Biostatistics (Main), All India Institute of Medical Sciences, New Delhi, India

**Keywords:** Epidemiology, Health policy, Applied mathematics, Statistics

## Abstract

Modelling is a well-established concept for understanding the typical shape and pattern of age-specific fertility. The distribution of India’s age-specific fertility rate (ASFR) is unimodal and positively skewed and is distinct from the ASFR of the developed countries. The existing models (P-K model, Gompertz model, Skew-normal model and G-P model considered here) that were developed, based on the experiences of the developed countries, failed to fit the single-year age-specific fertility pattern for India as a whole and for the six selected states. Our study has proposed four flexible models, to capture the diverse age pattern of fertility, observed in the Indian states. The proposed models were compared in three ways; among themselves, with the original models and with the popular Hadwiger model. The parameters of these proposed models were estimated through the Non-Linear Least Squares Method. To find the model with best fit, we used the corrected version of Akaike’s Information Criterion (AICc). Optimization of the four original models was successfully done. When the model was fitted to the empirical data of the 4th round of the National Family Health Survey conducted in 2015–2016, the results of this study showed that all the four proposed models outperform their corresponding original models and the Hadwiger model. When comparison among the proposed models was done, the Modified Gompertz Model provided the best fit for India, Uttar Pradesh and Gujarat. Whereas, the Modified P-K model gave the best fit for West Bengal, Tripura and Karnataka. The Modified G-P model is the most suitable model for Punjab. Although our proposed models illustrated the fitting of ASFR for India as a whole and the selected six states only, it provides an important tool for the policymakers and the government authorities to project fertility rates and to understand the fertility transitions in India and various other states.

## Introduction

The optimal way of unfolding the fertility and determining its curve through modeling, has been a crucial issue in the demographers’ research. Modelling fertility behaviour is not only practicable in estimating fertility indicators such as the General fertility rate (GFR), Total fertility rate (TFR), Age-specific fertility rate (ASFR), but also beneficial for forecasting and fertility projection that facilitate the population projections. In literature, there are a variety of parametric fertility models to explain the behaviour of the fertility pattern. The class of models with a certain built-in probability density function includes the Hadwiger model^1,2^, Hadwiger mixture model^3^, Coale–Trussell model^4–6^, Beta and Gamma models^6^, Pearson Type 1 curve^7,8^ and Type III curves^9^, P-K model and P-K mixture model^10^, Flexible Generalised skew-Normal model^11^, The scaled Weibull mixture model^12^, logistic model^13^, Skew logistic model^7,14^. Other researchers have designed their parametric models using mathematical functions. These include the Gompertz model^15–21^ and the adjusted error model^22^. All the fertility models have their advantages and work as best fit under different conditions.

**Figure 1:**
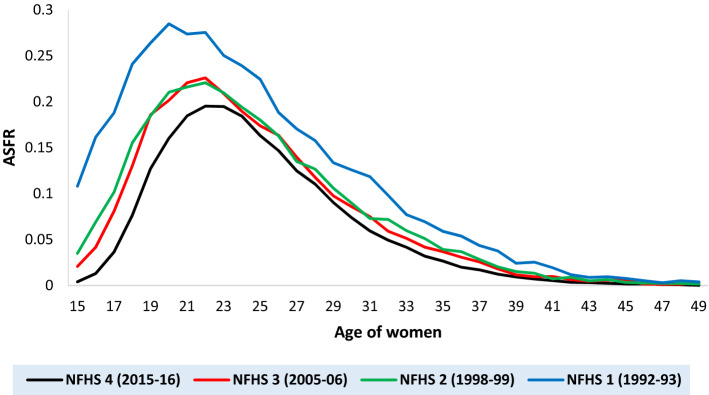
The age-specific fertility rate (ASFR) of India as per different National Family Health Survey (NFHS) rounds.

Over the years, it has been observed that the shape of the ASFR curve of the developing and the developed countries has changed significantly. India has a moderate level of fertility and an Early-peak type fertility schedule (when peak fertility is observed among women of the age group 20–24 years^23^). A rightward shift in the age of extreme fertility and a downward shift in the magnitude of extreme fertility has been observed in Indian ASFR (Fig. 1). The contribution of the adolescents’ fertility (fertility among women aged 15–19 years) in the total fertility dropped from 24 percent (NFHS-1^24^) to 12 percent (NFHS-4^27^). However, the overall structure of the Indian fertility schedule is unaltered over time. It is pertinent to mention that the pattern and the level of fertility in the developed countries are very different from the developing countries like India. In recent years, ASFR patterns of developed countries like the United Kingdom, the United States, and Ireland experienced one more bulge for the early age fertility apart from the extreme fertility. Chandola et al.^3^ conjectured that the differences in the fertility curve of the modern population might be linked to a mixture of two sub-populations (marital and non-marital), having different fertility patterns. Peristera and Kostaki^10^ also found this aberration pattern in early births in the other European countries. To account for such heterogeneity, Peristera and Kostaki^10^, fit their model on order-specific fertility rates. For the United States population, they fit separate models for the Black and the White population. Mixed models can be used to model this fertility pattern. Hadwiger mixture model^3^, The P-K mixed model^10^, the flexible generalized skew-normal model^11^, and the scaled Weibull mixed model^12^ will provide a better fit for these type of fertility patterns.

The other classes of fertility models are non-parametric. Polynomial model^28–30^, Spline model^31–35^ and the other models^36^ are non-parametric models of the age-specific fertility schedules. Although polynomial models are simple and easy to handle, they are not quite popular due to the non-interpretability of their parameters. On the other hand, the splines model requires the estimation of more number of parameters, which is unclear.

In recent literature, there have been only a handful of studies^7,28,29^, that attempt to fit the age-specific fertility pattern in the Indian context. To the best of our knowledge, no study has ever tried to fit a single-year, age-specific Indian fertility pattern, through parametric models. The existing parametric models that were developed based on the experiences of the developed countries, failed to fit the single-year ASFR of India as a whole and the six selected states. This provides a rationale for the present study. This study proposes four flexible models to capture the diverse fertility pattern observed in the Indian states. Evaluation of these models is done by fitting the ASFR curve of the six selected Indian states and the performance of each model is also measured by applying these models on the real data.

## Data and methodology

The present study used the data from the National Family Health Survey (NFHS-4), conducted in 2015–2016. The NFHS is a national representative cross-sectional survey conducted for the fourth time in India, as part of The Demographic Health Survey (DHS) Program. Previously, three rounds (1992–1993^24^, 1998–1999^25^ and 2004–2005^26^) have been successfully conducted. The International Institute of Population Sciences (IIPS) conducts the NFHS, with the collaborative efforts of a large group of organizations under the stewardship of the Ministry of Health and Family Welfare (MoHFW), Government of India. NFHS provides major indicators like mortality, fertility, maternal and child health indicators, family planning, child nutrition, domestic violence, morbidity estimates for several diseases etc. at the national and the state level. This is for the first time, that the NFHS-4 has given estimates of major indicators for 640 districts of India (as per the 2011 Census of India). NFHS-4 also provides information on several new and emerging issues, including insurance coverage, use of mosquito nets for malaria prevention, abortion, HIV testing during antenatal care, ownership of physical and economic assets by women, and domestic violence during pregnancy. The study sample for NFHS-4 is based on the stratified two-stage sampling of households. A total of 6, 99,686 eligible women of the age-group 15–49 years and 1, 12,122 eligible men of the age-group 15–54 years, were successfully interviewed using the Computer Assisted Personal Interviewing method. For more details regarding the survey design and the questionnaires, one can refer to the NFHS- 4 report^27^.

Amongst all the fertility indicators, the ASFR secures a crucial place because of its role in forecasting the population by the cohort component projection method. Though, the ASFR estimated from the empirical data is a good portrayal of the existing fertility behaviour of the population, using it for the projection of future population requires an afterthought. Owing to its ability, to pragmatically translate the assumptions about the future fertility behaviour, modeling of ASFR is of considerable interest to the demographers. If someone wants to analyse the mean age of childbearing or wants to know the proportion of births by mothers belonging to the adolescent age group (15–19) years or by mothers over the age of 35 years, information cannot be directly obtained through ASFR summary measure. There is also much information about the variance, kurtosis, and skewness of the ASFR curve of a particular region. Modelling would be necessary for those conditions. Modelling of age-specific fertility is also necessary to understand the phenomenon of age specific fertility transition among different countries. The foremost aim of any model building is to extract all possible information from the available data and to give an accurate picture of all the known and the unknown facets of the phenomena under study^22^. But, the approximation errors of the fertility model, serve as the primary drivers of deviation in the forecasted population^37^. Hence, it is necessary to identify the correct model for fertility schedule.

In order to simulate the fertility schedule, information on the birth histories of women in the age group of 15–49, is extracted from the NFHS-4. Although 5-year ASFR is reported in the national as well as state reports of NFHS-4, we have computed single-year ASFR for the modelling fertility pattern. The reference period for the calculation of single-year ASFR is 3 years before the survey, i.e., only the births that have occurred within 3 years, before the survey date was utilized to compute single-year ASFR. A total of 6, 98,185 eligible women were considered in our study. For the calculation of all India and state level single-year ASFR, we used STATA tfr2 module as described by Schoumaker^38^.

To find the true nature of the fertility schedule, it is better to choose a single year ASFR than 5-year grouped ASFR. However, single-year ASFR suffers from some fluctuations and some digit preferences at certain ages. To detect the correct trend of fertility patterns suffering from the problem of fluctuations, we have applied the smoothing technique. Here, we used *loess *(*locally estimated scatterplot smoothing*) techniques to smoothen the scatterplot. The advantage of loess smoothing is that it is a bit more flexible than the other smoothing techniques. It does not assume any particular mathematical form, to smoothen the data but allows them to discover the desired form from data itself. We have implemented loess ( ) function in R with 0.2 as the value of the smoothing parameter (20% smoothening). The reason for choosing the parameter equal to 0.2 is that, it is neither too small to eliminate fluctuations in the data nor too large to over-smoothen the scatter plot. In this study, we try to fit the fertility pattern of India and the 6 selected Indian states with four proposed models. For this purpose, based on the socio-cultural similarities, we have classified the Indian states into six geographical regions, depending on their locations; north, central, east, northeast, west and south. And from each of these six regions, one representative state was selected randomly (using random number generation). The region wise classification of states and the 6 selected states from their respective region is given in Table 1. The age-specific fertility pattern of India and the six selected states (Punjab, Uttar Pradesh, West Bengal, Tripura, Gujarat and Karnataka) are obtained from NFHS-4.

**Table 1:** Regional classification of Indian states.

Region	Name of states	Randomly selected states
North	Haryana, Himachal Pradesh, Jammu and Kashmir, Punjab, Uttarakhand	Punjab
Central	Chattisgarh, Madhya Pradesh, Uttar Pradesh	Uttar Pradesh
East	Bihar, Jharkhand, Odisha, West Bengal	West Bengal
Northeast	Arunachal Pradesh, Assam, Manipur, Meghalaya, Mizoram, Nagaland, Sikkim, Tripura	Tripura
West	Rajasthan, Gujarat, Maharashtra, Goa	Gujarat
South	Andhra Pradesh, Karnataka, Kerala, Tamil Nadu, Telangana	Karnataka

In order to evaluate our proposed model in comparison to the Hadwiger model, we have fitted Hadwiger model to the empirical fertility of India and the selected states and them compared the estimates. The functional form of the Hadwiger model is as follows:

$$f(x,\;a,\;b,\;c) = \frac{ab}{c}\left( \frac{c}{x} \right)^{\frac{3}{2}} \exp \left\{ { - b^{2} \left( {\frac{c}{x} + \frac{x}{c} - 2} \right)} \right\}$$

Here, *f*(.) is the ASFR at age *x* and *a, b, c* are the three parameters. Chandola et al.^3^ has discussed the demographic interpretation of these parameters. Parameter *a* is related to the total fertility, *b* is associated with the height of the ASFR curve and parameter *c* is associated with the mean age of childbearing whereas the term $\frac{ab}{c}$ is associated with the extent of extreme fertility.

### Proposed models

#### Model 1 (Modified P-K Model)

The first model is an extension of the work done by Peristera and Kostaki^10^, in which a constant parameter *a* is added to capture the age specific fertility in the later ages (45–49 age-group). The original P-K Model^10^ is as follows:

$$f(x,\;b,\;\mu ,\;\sigma_{1} ,\;\sigma_{2} ) = \left\{ {\begin{array}{*{20}c} {b \exp \left( {\frac{x - \mu }{{\sigma_{1} }}} \right)^{2} } & {\quad if\; x \le \mu } \\ {b \exp \left( {\frac{x - \mu }{{\sigma_{2} }}} \right)^{2} } & {\quad if \;x > \mu } \\ \end{array} } \right.$$

Here *f*(.) is the ASFR of women of age *x*. *b* is the peaked value of fertility, μ is the age at the time of peaked fertility, $\sigma_{1}$ and $\sigma_{2}$ depicts the spread of the ASFR curve, before and after the peaked fertility respectively. A Similar concept was used by Gayawan et al.^22^ to fit the ASFR of some African countries and it was referred to as the *Adjusted Error Model* (AEM). In this model, a constant term is added to the complementary error function.

Our proposed model is defined as follows:

$$f(x,\;a,\;b,\;\mu ,\;\sigma_{1} ,\;\sigma_{2} ) = \left\{ {\begin{array}{*{20}c} {a + b \exp \left( {\frac{x - \mu }{{\sigma_{1} }}} \right)^{2} } & {\quad if\; x \le \mu } \\ {a + b \exp \left( {\frac{x - \mu }{{\sigma_{2} }}} \right)^{2} } & {\quad if\; x > \mu } \\ \end{array} } \right.$$

In our model, *a* handles fertility in the late ages of the reproductive span. All the other parameters have the same interpretation as explained above. If we put *a* = *0* in the Modified P-K Model, it reduces to a model proposed by Peristera and Kostaki^10^.

#### Model 2 (Modified Gompertz Model)

The second model that is proposed is an extension of Gompertz model, in which we have added one scale parameter $\gamma$. The functional form of Gompertz model is taken from Hoem et al.^6^ and Mathivanan et al.^39^, which is given as follows:

$$f(x,\;\alpha ,\;\beta ,\;m) = \left( {\frac{\alpha }{\beta }} \right) \exp \left[ { - \left( {\frac{x - m}{\beta }} \right) - \alpha \exp \left\{ { - \left( {\frac{x - m}{\beta }} \right)} \right\}} \right]$$

The three parameter Gompertz model is not quite flexible. Murphy and Nagur^20^ showed that it has fixed kurtosis and therefore, it does not fit well to India as well as to the states’ ASFR curves. After inclusion of scale parameter $\gamma$, the curve fits very well. The proposed model is defined below:

$$f(x,\;\alpha ,\;\beta ,\;\gamma ,\;m) = \left( {\frac{\alpha }{\beta }} \right)^{\gamma } \exp \left[ { - \left( {\frac{x - m}{\beta }} \right) - \alpha \exp \left\{ { - \left( {\frac{x - m}{\beta }} \right)} \right\}} \right]$$

*m* is defined as the lowest marriage age of women in the population. Our model will reduce to Gompertz model as soon as we put $\gamma$ = *1* in the above model.

#### Model 3 (Modified Skew Normal Model)

The third model presented here is a modification of the generalized skew normal function. The original skew normal distribution is defined as follows:

$$f(x,\;\lambda ,\;\sigma^{2} ,\;\delta ) = 2\sigma^{ - 1} \phi \left( {\frac{x - \lambda }{\sigma }} \right)\Phi \left\{ {\delta \left( {\frac{x - \lambda }{\sigma }} \right)} \right\}$$

where ϕ and Φ are the density and the distribution function of the standard normal distribution respectively. *λ* is the location parameter and *σ* is the scale parameter. *δ* is the skewness parameter. The detailed property of this function was first studied by Azzalini^40^. In the modified version of this model, the skewness of the ASFR curve is controlled by *δ* and the two scale parameters $s_{1}$ and $s_{2}$.The proposed model is defined as follows:

$$f(x,\;\theta ,\;\lambda ,\;s_{1} ,\;s_{2} ,\;\delta ) = \left\{ {\begin{array}{*{20}c} {\theta \exp \left( { - \frac{1}{2}\left( {\frac{x - \lambda }{{s_{1} }}} \right)^{2} } \right) \Phi \left( {\frac{\delta x - \lambda }{{s_{1} }}} \right)} & {\quad if\; x \le \lambda } \\ {\theta \exp \left( { - \frac{1}{2}\left( {\frac{x - \lambda }{{s_{2} }}} \right)^{2} } \right) \Phi \left( {\frac{\delta x - \lambda }{{s_{2} }}} \right) } & {\quad if\; x > \lambda } \\ \end{array} } \right.$$

Here, θ reflects the peaked value of the age specific fertility, *λ* being the age at which the peaked fertility is being achieved. $s_{1}$ and $s_{2}$ represents the deviation of the ASFR curve, before and after the peaked fertility respectively.

#### Model 4 (Modified G-P Model):

The fourth model that we have proposed is a variation of the behavioral model, suggested by Gupta and Pasupuleti^41^. The original form of G-P Model^24^ is as follows:

$$f(x, \;p, \;q,\;r) = p x^{q} \left( {1 - \frac{x}{49}} \right)^{r}$$

Here, *f*(.) is the ASFR at age x, parameter *q* being responsible for the amplification in the level of the fertility, due to the ‘exposure to marriage and sexual union’. Parameter *r* is responsible for the decline in the level of fertility due to the ‘birth control measures and the biological inability to reproduce’. This model overestimates the early age specific fertility and underestimates peak age specific fertility. Hence, we have introduced a constant parameter *s,* to capture the late age specific fertility and to make few scale and location modifications to the variable *x*. The proposed model is expressed as follows:

$$f(x, \;p,\;q,\;r,\;s) = s + p\left( {\frac{x - 15}{{49}}} \right)^{q} \left( {1 - \frac{{\left( {x - 15} \right)}}{49}} \right)^{r}$$

### Estimation of parameters of the models

For the estimation of the proposed models, we have the applied Non-Linear Least Squares Method. Under this method, we have minimized the sum of squares due to error (SSE), with respect to the parameters of the model. SSE function is given by:

$$SSE = \mathop \sum \limits_{x} \left( {f(x) - \widehat{f(x)}} \right)^{2}$$

Here *f*(*x*) is the observed ASFR at age *x* and $\widehat{f(x)}$ is the estimated ASFR from the proposed models. We have used *nlminb*( ) function in R, for non-linear minimization of SSE function. *nlminb*( ) function estimates the parameters, along with their asymptotic standard errors. In this study, we did not report the asymptotic standard errors as per the convention^10^.

### Performance of the models

While modelling the ASFR, it is necessary to check the performance of different models. Usually, one can plot the predicted values and the observed values of the ASFR from the model and then choose the best fit among all the models. In quantitative terms, SSE should be calculated for each model and the one with the least SSE will be the best fit model. However, this will not take care of the model complexity, resulting from the inclusion of more parameters. Hence in order to keep the model fit and to consider its complexity, we have used corrected Akaike’s Information Criterion (AICc)^42^, to select the best performing model in this study. AICc is Akaike’s Information Criterion (AIC)^43^ adjusted for small sample sizes. AIC for a particular model adjusts the SSE, with the number of parameters and the number of observations in the model. The formula of AICc is as follows:

$$AIC = 2k + n \ln \left( {\frac{SSE}{{n - k}}} \right)$$

$$AICc = AIC + \frac{2k(k + 1)}{{(n - k - 1)}}$$

Here, *k* denotes the number of parameters present in the model and *n* denotes the number of observations. As $n \to \infty$, the results of the AICc converge to AIC. The criteria is to choose the model with the least value of AICc, as the best performing model.

## Results

### Age-specific fertility schedule of India and six selected states

**Figure 2:**
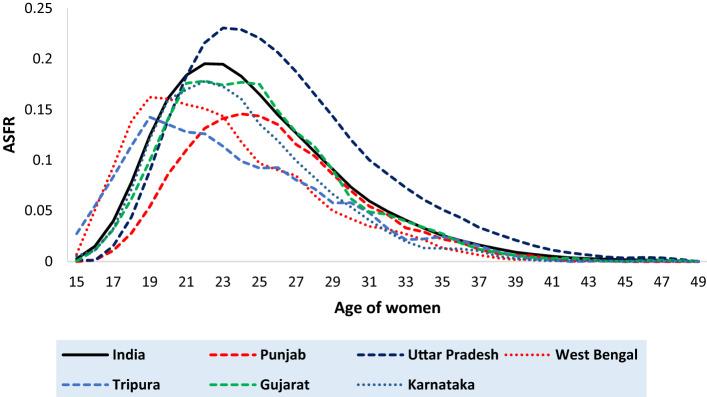
Age-specific fertility rate (ASFR) of India and the 6 selected states as per Fourth round of National Family Health Survey (NFHS).

Figure 2 shows the single year ASFR curves for India and the six selected states (Punjab, Uttar Pradesh, West Bengal, Tripura, Gujarat and Karnataka). All the ASFR curves are unimodal and skewed towards the right (positively skewed), i.e. age-specific fertility rises fast in the initial years, attains its peak in the middle ages (19–24) and then falls slowly towards the higher age of the reproductive period. The selected states have different levels of fertility at various ages. Hence, the Total Fertility Rate (TFR) also differs. Here, Uttar Pradesh has the highest level of TFR (2.75) followed by Gujarat (2.03), Karnataka (1.79), West Bengal (1.76), Tripura (1.68) and Punjab (1.60). The value of the peak fertility and its attainment age also varies over selected states. The ASFR curve of India attains its peak fertility (0.195) at the age of 22. Among the selected states, Uttar Pradesh reaches its peak fertility (0.230) at the age of 23, followed by Karnataka (0.178 at the age of 22), West Bengal (0.162 at the age of 19), Punjab (0.146 at the age of 24) and Tripura (0.143 at the age of 19). However, the peak of the fertility pattern of Gujarat remains somewhat constant between 21 to 25 years. Tripura has the highest age-specific fertility at the early ages, whereas, Punjab has the lowest level of fertility at the early ages.

Uttar Pradesh has the highest level of fertility in the later ages (45–49 years), while Punjab has the lowest level of fertility in the later ages too. It is also observed from the figure, that although Karnataka and West Bengal have approximately the same level of TFR, their ASFR is very much different in shape. From the age of 15–20 years, West Bengal has a higher level of fertility than that of Karnataka; in the later ages, Karnataka has a higher level of fertility than that of West Bengal. The rate of increase from the start of the curve to its peak is highest for West Bengal and least for Punjab.

### Performance of the proposed models with respect to the original models

Table 2 displays the performance of the Modified P-K Model vs. P-K model in terms of SSE and AICc values for India and the six selected states. The P-K model has 4 parameters, whereas the Modified P-K Model has five parameters. By comparing SSE and AICc values of both the models, we have found that the Modified P-K Model is working better only for India, Uttar Pradesh and Gujarat. The SSE values of both the models of Tripura are approximately the same. But the P-K Model is preferred over the Modified P-K Model, because it has less number of parameters. Likewise, Table 3 shows the performance of the Modified Gompertz Model vs. Gompertz model, w.r.t their SSE and AICc values. In comparison to the Gompertz model, both the SSE and AICc values under the Modified Gompertz Model are smaller. It means that the Modified Gompertz Model is more flexible and is able to describe the empirical fertility schedules of India and the 6 selected states. Tables 4 and 5 shows the performance of the Modified Skew Normal Model and the Modified G-P Model vs. Skew-Normal model and G-P Model, respectively. The Modified Skew Normal Model has the least SSE and AICc values, as compared to the Skew-Normal model for India and all the other selected states (Table 4), i.e. the Modified Skew Normal Model outperforms the Skew-Normal model, in recreating the fertility schedules. From the results in Table 5, it can be seen that the Modified G-P Model has the least SSE and AICc value, as compared to the G-P model in India and all the six selected states. It means that the Modified G-P Model provides better results than the G-P model, for fitting the fertility patterns of India and the selected states. Figures B1 to B4 in the Appendix B, show the observed and the fitted age-specific fertility pattern, under the proposed model and the existing model under study. We have shown these graphs only for Indian fertility schedules.

**Table 2:** Minimized sum of square (SSE) and AICc values under P-K Model and Modified P-K Model for India and the 6 selected states.

Model	Region	SSE	AICc
P-K Model (K = 4)	India	0.00056	− 372.633
	Punjab	0.00032	− **392.736**
	Uttar Pradesh	0.00085	− 358.236
	West Bengal	0.00061	− **369.945**
	Tripura	0.00061	− **370.204**
	Gujarat	0.00091	− 356.094
	Karnataka	0.00031	− **393.476**
Modified P-K Model (K = 5)	India	0.00028	− **393.008**
	Punjab	0.00046	− 366.255
	Uttar Pradesh	0.00032	− **389.052**
	West Bengal	0.00074	− 359.33
	Tripura	0.00066	− 366.825
	Gujarat	0.00066	− **366.374**
	Karnataka	0.00086	− 353.947

The least value of AICc is written in the bold case for each state.

**Table 3:** Minimized sum of square (SSE) and AICc values under Gompertz Model and Modified Gompertz model for India and the 6 selected states.

Model	Region	SSE	AICc
Gompertz Model (K = 3)	India	0.0684	− 208.41
	Punjab	0.01907	− 253.122
	Uttar Pradesh	0.14701	− 181.63
	West Bengal	0.0304	− 236.79
	Tripura	0.02212	− 247.924
	Gujarat	0.05463	− 216.278
	Karnataka	0.03432	− 232.544
Modified Gompertz Model (K = 4)	India	0.00034	− **390.9**
	Punjab	0.00017	− **413.885**
	Uttar Pradesh	0.00022	− **405.284**
	West Bengal	0.00054	− **374.085**
	Tripura	0.00155	− **337.176**
	Gujarat	0.0016	− **336.202**
	Karnataka	0.00075	− **362.856**

The least value of AICc is written in the bold case for each state.

**Table 4:** Minimized sum of square (SSE) and AICc values under Skew Normal Model and Modified Skew Normal Model for India and 6 selected states.

Model	Region	SSE	AICc
Skew Normal Model (K = 3)	India	0.00449	− 303.731
	Punjab	0.02743	− 240.39
	Uttar Pradesh	0.00241	− 325.443
	West Bengal	0.01739	− 256.334
	Tripura	0.01009	− 275.384
	Gujarat	0.00968	− 276.829
	Karnataka	0.02038	− 250.795
Modified Skew Normal Model (K = 5)	India	0.00029	− **392.215**
	Punjab	0.00056	− **368.75**
	Uttar Pradesh	0.00032	− **388.852**
	West Bengal	0.00085	− **354.352**
	Tripura	0.00061	− **36.061**
	Gujarat	0.00061	− **36.321**
	Karnataka	0.00084	− **354.882**

The least value of AICc is written in the bold case for each state.

**Table 5:** Minimized sum of square (SSE) and AICc values under G-P Model and Modified G-P Model for India and 6 selected states.

Model	Region	SSE	AICc
G-P Model (K = 3)	India	0.00718	− 287.307
	Punjab	0.01829	− 254.579
	Uttar Pradesh	0.01326	− 265.841
	West Bengal	0.00963	− 277.032
	Tripura	0.00472	− 302.004
	Gujarat	0.00788	− 284.046
	Karnataka	0.00721	− 287.162
Modified G-P Model (K = 4)	India	0.00038	− **386.121**
	Punjab	0.00039	− **385.583**
	Uttar Pradesh	0.00015	− **419.288**
	West Bengal	0.00159	− **336.338**
	Tripura	0.00056	− **372.685**
	Gujarat	0.00153	− **337.806**
	Karnataka	0.00081	− **359.998**

The least value of AICc is written in the bold case for each state.

**Figure 3:**
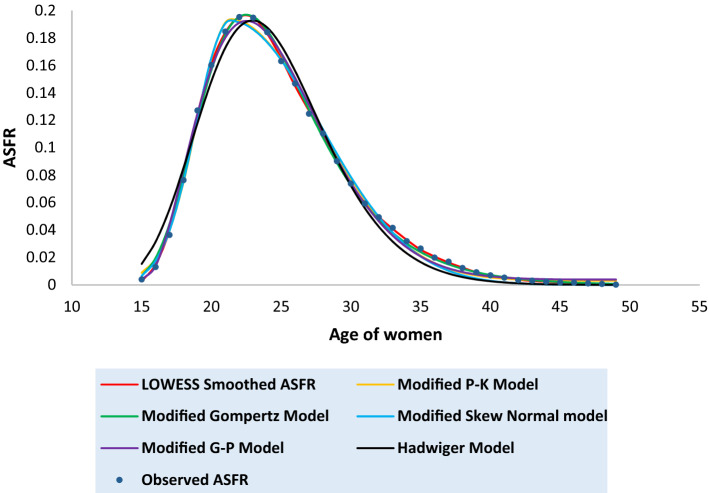
Observed and expected age-specific fertility rate (ASFR) for India under different proposed models and Hadwiger model.

**Figure 4:**
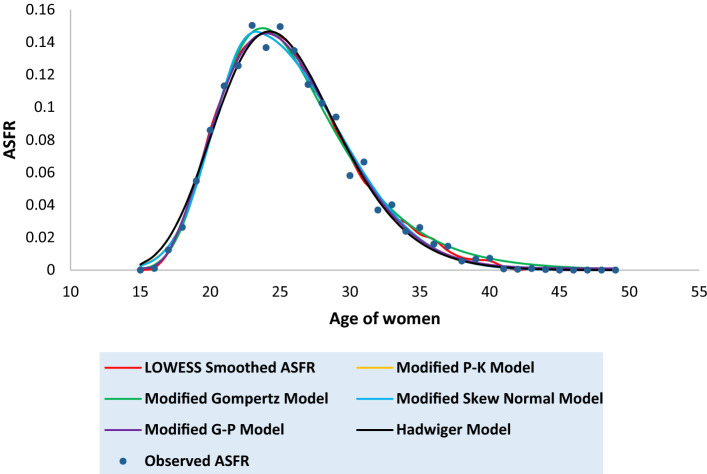
Observed and expected age-specific fertility rate (ASFR) for Punjab under different proposed models and Hadwiger model.

**Figure 5:**
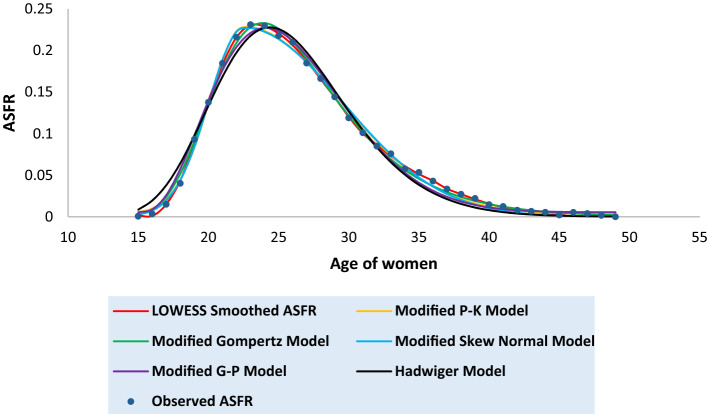
Observed and expected age-specific fertility rate (ASFR) for Uttar Pradesh under different proposed models and Hadwiger model.

**Figure 6:**
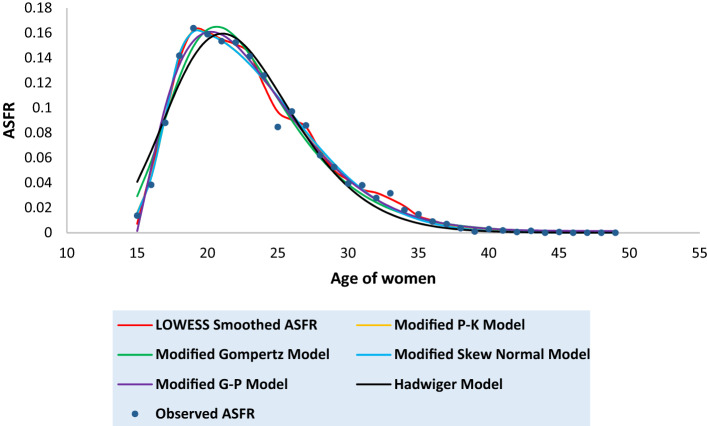
Observed and expected age-specific fertility rate (ASFR) for West Bengal under different proposed models and Hadwiger model.

**Figure 7:**
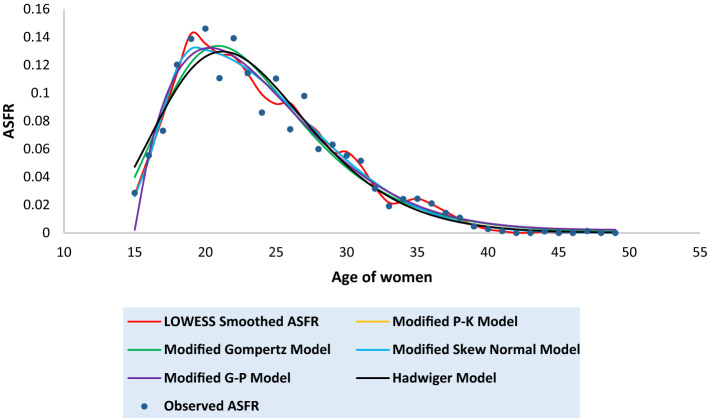
Observed and expected age-specific fertility rate (ASFR) for Tripura under different proposed models and Hadwiger model.

**Figure 8:**
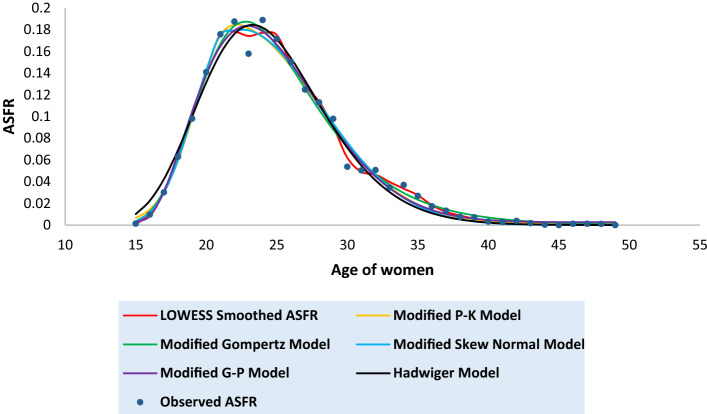
Observed and expected age-specific fertility rate (ASFR) for Gujarat under different proposed models and Hadwiger model.

**Figure 9:**
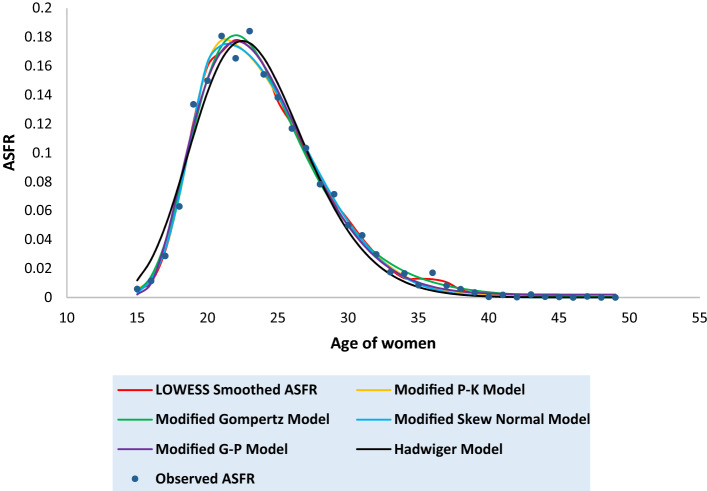
Observed and expected age-specific fertility rate (ASFR) for Karnataka under different proposed models and Hadwiger model.

Tables A1 to A7 in the Appendix A present the observed and the fitted estimates of fertility pattern, under each of the four proposed models and Hadwiger model. The tables also present the number of parameters, estimated value of TFR, the values of SSE, the values of AICc and the value of coefficient of determination (${\text{R}}^{2}$) for each model. The least value of AICc is written in bold case for each case. Figures 3, 4, 5, 6, 7, 8 and 9 show the smoothened observed and fitted curve, under the proposed models and the Hadwiger model for India, Punjab, Uttar Pradesh, West Bengal, Tripura, Gujarat and Karnataka respectively.

India’s fertility pattern is best predicted by the Modified Gompertz Model, as it provides the best fit among all the other models under comparison (Table A1 and Fig. 3). The Modified Gompertz Model also gives the best fit for the fertility pattern of Uttar Pradesh (Table A3 and Fig. 5) and Gujarat (Table A6 and Fig. 8). The Modified P-K Model is superior to the other models under comparison, for the fertility schedules of West Bengal (Table A4 and Fig. 6), Tripura (Table A5 and Fig. 7) and Karnataka (Table A7 and Fig. 9). On the other hand, the fertility pattern of Punjab is best fitted by the the Modified G-P Model amongst all the other models (Table A2 and Fig. 4). From Tables A1 to A7, we observe that the estimated values of single-year ASFR under the Modified P-K Model and the Modified Skew Normal Model are quite close. Both have five parameters. However, the AICc values of the Modified P-K Model are slightly smaller than that of the Modified Skew Normal Model for India and for each of the selected states. Further, Tables 6, 7, 8, 9 and 10 display the estimates of parameters of the Modified P-K Model, the Modified Gompertz Model, the Modified Skew Normal Model, the Modified G-P Model and Hadwiger model respectively.

**Table 6:** Estimates of parameters of Modified P-K Model for India and 6 selected states.

Region	Modified P-K Model				
	*b*	*μ*	*σ*_1_	*σ*_2_	*a*
India	0.1902	21.3593	3.4055	8.9152	0.00307
Punjab	0.1457	23.0807	3.9941	8.3289	0.00039
Uttar Pradesh	0.2252	22.5390	3.4817	9.7071	0.00338
West Bengal	0.1605	18.8874	2.5272	9.6826	0.00104
Tripura	0.1315	19.0725	3.1982	11.300	0.00032
Gujarat	0.1827	21.8334	3.5650	8.5596	0.00190
Karnataka	0.1764	20.9251	3.0144	8.1487	0.00144

**Table 7:** Estimates of parameters of Modified Gompertz Model for India and 6 selected states.

Region	Modified Gompertz Model			
	*α*	*β*	*γ*	*m*
India	4.3619	4.0885	13.1039	16.4648
Punjab	4.2900	4.0528	9.67184	17.8301
Uttar Pradesh	4.7330	4.3582	13.316	17.0962
West Bengal	4.1874	3.9694	11.7965	14.966
Tripura	5.1183	4.7769	8.99602	13.1106
Gujarat	4.2689	4.0249	13.2025	16.9462
Karnataka	3.8511	3.698	13.2892	17.0609

**Table 8:** Estimates of parameters of Modified Skew Normal Model for India and 6 selected states.

Region	Modified Skew Normal Model				
	*λ*	*s*_1_	*δ*	*s*_2_	*θ*
India	21.3305	2.4382	2.8960	6.4817	0.1924
Punjab	23.0761	2.8307	2.4989	5.9163	0.1460
Uttar Pradesh	22.5175	2.4965	2.9761	7.0375	0.2275
West Bengal	18.8777	1.7939	3.3029	6.9201	0.1612
Tripura	19.0693	2.2651	3.5001	8.0211	0.1318
Gujarat	21.9311	2.6643	1.4815	6.0231	0.1864
Karnataka	20.9544	2.1678	1.5237	5.7689	0.1793

**Table 9:** Estimates of parameters of Modified G-P Model for India and 6 selected states.

Region	Modified G-P Model			
	*p*	*q*	*r*	*s*
India	165.321	2.426	13.3747	0.00392
Punjab	837.923	3.3364	14.8441	0.00110
Uttar Pradesh	492.428	2.9784	13.0964	0.00544
West Bengal	9.42817	1.2826	10.7123	0.00141
Tripura	2.98192	0.9902	8.12041	0.00223
Gujarat	321.995	2.7327	14.2034	0.00263
Karnataka	359.998	2.6692	15.7946	0.00202

**Table 10:** Estimates of parameters of Hadwiger Model for India and 6 selected states.

Region	Hadwiger Model		
	*a*	*b*	*c*
India	1.2141	3.6940	24.2482
Punjab	0.9044	3.9764	25.4145
Uttar Pradesh	1.5322	3.6763	25.7625
West Bengal	1.0012	3.4104	22.4573
Tripura	0.9832	2.8627	23.2429
Gujarat	1.1338	3.8335	24.4840
Karnataka	1.0023	4.0238	23.5473

We have also explored the possible demographic interpretation of the new parameters added in the proposed models. There is a strong association between the later ages (45–49 years) fertility and the constant parameter *a* of the Modified P-K Model. The correlation coefficient between these two (r = 0.857) are significant. We have also reviewed the demographic interpretation of the parameters that were already present in the previously existing models. The parameter *b* in the Modified P-K Model is significantly related to the extent of peak fertility (r = 0.988). Parameter μ in the Modified P-K Model is related to the women’s age at extreme fertility, whereas $\sigma_{1}$ and $\sigma_{2}$ describes the spread of the ASFR curve before and after the peaked fertility respectively. All the fertility schedules are skewed towards the right. For the Modified Gompertz Model, we find that parameter γ is strongly related to the extent of extreme fertility and the term is significantly correlated (r = 0.998) to the total fertility rate. Notwithstanding, we could not test for the association of parameter *m* with the lowest age of childbearing in the Modified Gompertz Model. There is a great resemblance between the parameters θ and λ from the Modified Skew Normal Model and *b* and μ from the Modified P-K Model. Both the estimates are quite close and hence have similar interpretations. θ is strongly associated with level of extreme fertility (r = 0.985) whereas λ is related to the age at modal fertility. The parameters $s_{1}$ and $s_{2}$ of the Modified Skew Normal Model represents the spread of the ASFR curve. Estimates of the parameter δ in each case was found to be positive under the Modified Skew Normal Model. It means that ASFR curve is positively skewed. The parameter *s* in the Modified G-P Model is found to be significantly correlated (r = 0.969) with the level of fertility in the later ages (45–49 years). Parameter *q* is strongly associated with the age of women at extreme fertility (r = 0.968). However, we could not find reasonable interpretations for the parameters *p* and *r* of the Modified G-P Model. Further, parameter *a* in Hadwiger model is significantly associated with the total fertility (r = 0.997). The other parameter c in Hadwiger model is closely related to the mean age of childbearing However, the interpretation of the parameter *b* in the Hadwiger model as given by Chandola et al.^10^ could not be justified in terms of the height of the fertility curve.

## Discussion

India is a vast country comprising 28 states and 8 union territories, with wide demographic and socio-cultural diversity. Many of the Indian states have higher population than several countries of the world. Comparing the fertility schedules of the Indian states is an extensive exercise. In comparison to the previous studies^7,29^ done on the modelling of Indian fertility schedule, our study paid more attention to model with the single-year ASFR of India and the six states. Our study is also distinct from the Pandey et al.^28^, as it proposes four parametric models for single-year ASFR. Pandey et al.^28^ also fitted the data of single-year ASFR through polynomial functions.

Results of our study conclude that the Modified P-K Model performs better than P-K model only if the tail of the fertility curve has some amount of fertility. P-K model provides best fit for the countries with non-enhanced early-age fertility^10^. P-K model performed better than Gamma model, Hadwiger model and quadratic spline model for the majority of countries (Norway, Denmark, Italy, Greece and Belgium) examined^10^. The Modified P-K Model generally fits well to the fertility schedules with a low level of fertility. It has provided the best fit to the fertility pattern of West Bengal (TFR = 1.8), Tripura (TFR = 1.7) and Karnataka (TFR = 1.8). The AICc value of the Modified Gompertz Model for Karnataka is quite close to that of the Modified P-K Model. The common feature of the fertility curve of West Bengal and Tripura is that, both have a high level of fertility in the early ages (15–19) and the Modified P-K Model gives a considerable amount of fertility in the early ages.

While an experiment of Hoem et al.^6^ to fit Gompertz curve to Danish fertility schedules for the calendar years 1962–1971 was found to be unpromising, the Modified Gompertz Model provided best fit for India (moderate level TFR = 2.2), Uttar Pradesh (High-level TFR = 2.8) and Gujarat (Low-level TFR = 2.0), i.e. it can reproduce all types of fertility schedules, where the total fertility is greater or equal to two. However, it is observed that it works better for those fertility curves, where the decline in the level of fertility after the peak is not so rapid.

Mazzuco and Scarpa showed that the skew-normal density is quite good for modelling the unimodal fertility curve. Skew-normal density has outperformed Gamma, Hadwiger and P-K model for the United States fertility data of 1963^11^. Our study has confirmed that the Modified Skew Normal Model has worked better than the original skew-normal model for India and all the other six states. Gupta and Pasupuleti have examined the applicability of G-P model on the cohort fertility schedules of India, Finland, Sweden, Netherlands, Switzerland and USA and the period fertility schedules of Bhutan, Iran, Somoa and Algeria^41^. The results of their study concluded that the fit was inferior to Gamma and Hadwiger model, but superior to the Gompertz model. However, the differences were marginal^41^. The Modified G-P Model provided better fitting than that of G-P model for the fertility schedule of India and all the six states considered in our study.

Although Punjab also has a low level of total fertility, the Modified G-P Model has provided the best fit. The reason behind the failure of the Modified P-K Model, for the best fit of Punjab might be that, the ASFR curve of Punjab has a shallow level of fertility in the early and the later ages. The inclusion of constant parameters in the Modified P-K Model and the Modified G-P Model, gives more weights of fertility in the tail of the fertility curve, when compared to the model without these constant parameters. Figures of the empirical and the observed fertility patterns, show that the Hadwiger model overestimates the early and the age-specific fertility around the peak. Though the attempt to fit the proposed models to the recent single-year ASFR curve has met some success, there is great scope of work for other researchers to find the suitability of these models for grouped and order-specific ASFR and the past fertility patterns of India and its varied states.

Most of the studies^7,10,16,22^ have either used AIC or residual sum of squares criterion to evaluate the performance of the proposed models. However, our study has used the corrected version of AIC criterion (AICc). Burnham and Anderson^44^ suggested that, AICc should be preferred over AIC when the ratio of the number of observations (*n*) and the number of parameters (*k*) is less than 40. In our study, the number of observations is 35 and the number of parameters is either 3, 4 or 5. So in each case, the ratio will be less than 40. Hence, the use of AICc is justified here. This also makes our study distinct from other studies.

It has been a subject of great discussion, whether the period or cohort ASFR should be adopted for the illustration of the model fit. Our results were based on the period fertility only. However, there is a lot of scope for researchers to fit our proposed models on cohort fertility. We have considered period data, since period data is easily available for analytical purpose and it also enables us to evaluate and examine the influence of recent demographic events and targeted interventions. On the other hand, cohort data either suffers from attrition or difficulty to reconstruct. Additionally, cohort data is concerned with the long term development, as cohort trends would be observable only if it is followed up for a longer period of time.

## Conclusion

The models that we have proposed are extensions to P-K Model, Gompertz model, generalized skew-normal function and Gupta and Pasupuleti model. These proposed models were compared among themselves and with their previously existing forms. Results of this study suggests, that all the proposed models are quite flexible and are able to produce a close fit to the variable type of the observed fertility pattern. It is pertinent to mention, that all the proposed models are more efficient than the original models considered in this article and the Hadwiger model, in terms of the goodness of fit for fertility schedules of India and its states. The demographic interpretation of the parameters of the model is also explored in this study. It is evident from the demographic interpretation of the parameters that the demographic profile of the Indian states is much more diversified. Although our proposed models have illustrated the fitting of fertility schedules of India on the whole and for the six states only, it was intended to provide an important channel, for the policymakers and the government authorities, to project the fertility rates and to understand the fertility transitions in India and its various states.

## Supplementary Information

Supplementary Information.

## Data Availability

The datasets analysed in our study are available in https://dhsprogram.com/data/available-datasets.cfm.

## Abbreviations

NFHS: National Family Health Survey
DHS: Demographic Health Survey
IIPS: International Institute of Population Sciences
MoHFW: Ministry of Health and Family Welfare
GFR: General fertility rate
TFR: Total fertility rate
ASFR: Age-specific fertility rate
SSE: Sum of square due to error
AIC: Akaike’s information criterion
AICc: Corrected version of Akaike’s information criterion
LOWESS: Locally estimated scatterplot smoothing
