# Supplementary material for: Experiments in modeling recent Indian fertility pattern
Source: Sci Rep. 2021 Mar 23;11:6592. doi: 10.1038/s41598-021-85959-z (PMC7987984; doi:10.1038/s41598-021-85959-z)
Supplement: Supplementary file 1 — Supplementary Information. [file 41598_2021_85959_MOESM1_ESM.docx]

**“Experiments in Modeling Recent Indian Fertility Pattern”**

*Ujjaval Srivastava^1,2,*^, Kaushalendra Kumar Singh^1^, Anjali Pandey^3^ and Neeraj Narayan^1^*

^1^ Department of Statistics, Banaras Hindu University, Varanasi, India.

^2^Ministry of Tourism, Government of India.

^3^ Department of Biostatistics (Main), All India Institute of Medical Sciences, New Delhi, India

**Remark to be printed as note:** The views expressed by Ujjaval Srivastava in this paper, are his own and not that of the Government of India**.**

* Corresponding Author (Email: [ujjavalsri777@gmail.com](mailto:ujjavalsri777@gmail.com))

**Appendix: A**

**Table A1: Observed, LOWESS smoothed and estimated values of ASFR (per 1000) for India along with the values of SSE and AICc under different proposed models**

| **Age** | **Observed ASFR (per 1000)** | **LOWESS Smoothed ASFR (per 1000)** | **Estimated ASFR (per 1000) (India)** | | | | |
| --- | --- | --- | --- | --- | --- | --- | --- |
|  |  |  | **Modified P-K Model** | **Modified Gompertz Model** | **Modified Skew Normal Model** | **Modified G-P Model** | **Hadwiger Model** |
| **15** | 4.059 | 2.689 | 8.892 | 6.507 | 6.611 | 3.921 | 15.361 |
| **16** | 13.092 | 15.032 | 19.061 | 19.737 | 17.629 | 13.855 | 31.530 |
| **17** | 36.460 | 39.834 | 40.036 | 44.624 | 39.730 | 44.222 | 55.354 |
| **18** | 76.410 | 78.168 | 74.988 | 80.157 | 75.676 | 84.774 | 85.334 |
| **19** | 127.167 | 124.265 | 120.825 | 120.247 | 121.827 | 125.038 | 117.945 |
| **20** | 160.223 | 161.312 | 165.338 | 156.655 | 165.758 | 158.032 | 148.603 |
| **21** | 184.591 | 183.994 | 191.257 | 182.745 | 190.612 | 180.297 | 172.977 |
| **22** | 195.206 | 195.328 | 192.384 | 195.516 | 191.349 | 191.081 | 188.072 |
| **23** | 194.729 | 194.864 | 187.027 | 195.482 | 186.096 | 191.405 | 192.735 |
| **24** | 184.244 | 182.993 | 177.380 | 185.353 | 176.731 | 183.255 | 187.570 |
| **25** | 163.084 | 165.094 | 164.135 | 168.601 | 163.889 | 168.980 | 174.451 |
| **26** | 146.746 | 144.908 | 148.198 | 148.456 | 148.405 | 150.882 | 155.884 |
| **27** | 124.741 | 126.796 | 130.588 | 127.433 | 131.224 | 130.977 | 134.437 |
| **28** | 110.344 | 108.589 | 112.331 | 107.227 | 113.302 | 110.876 | 112.333 |
| **29** | 90.255 | 91.121 | 94.362 | 88.828 | 95.527 | 91.756 | 91.247 |
| **30** | 73.864 | 73.519 | 77.453 | 72.692 | 78.646 | 74.390 | 72.261 |
| **31** | 59.359 | 59.740 | 62.169 | 58.920 | 63.226 | 59.207 | 55.932 |
| **32** | 49.383 | 49.535 | 48.859 | 47.401 | 49.633 | 46.360 | 42.407 |
| **33** | 41.652 | 40.833 | 37.665 | 37.911 | 38.046 | 35.806 | 31.555 |
| **34** | 31.917 | 32.833 | 28.559 | 30.182 | 28.478 | 27.369 | 23.084 |
| **35** | 26.517 | 25.726 | 21.383 | 23.942 | 20.815 | 20.796 | 16.626 |
| **36** | 19.951 | 20.714 | 15.900 | 18.939 | 14.856 | 15.801 | 11.805 |
| **37** | 16.948 | 16.315 | 11.836 | 14.948 | 10.354 | 12.098 | 8.274 |
| **38** | 12.367 | 12.647 | 8.910 | 11.778 | 7.046 | 9.420 | 5.730 |
| **39** | 9.254 | 9.239 | 6.864 | 9.268 | 4.682 | 7.529 | 3.924 |
| **40** | 7.035 | 7.053 | 5.474 | 7.285 | 3.038 | 6.229 | 2.661 |
| **41** | 5.326 | 5.162 | 4.555 | 5.721 | 1.925 | 5.358 | 1.787 |
| **42** | 3.515 | 3.769 | 3.965 | 4.490 | 1.191 | 4.790 | 1.190 |
| **43** | 3.120 | 2.947 | 3.596 | 3.523 | 0.720 | 4.430 | 0.786 |
| **44** | 2.381 | 2.383 | 3.372 | 2.762 | 0.425 | 4.210 | 0.515 |
| **45** | 1.769 | 1.886 | 3.239 | 2.165 | 0.245 | 4.079 | 0.335 |
| **46** | 1.723 | 1.593 | 3.162 | 1.697 | 0.138 | 4.004 | 0.217 |
| **47** | 1.172 | 1.203 | 3.119 | 1.330 | 0.076 | 3.963 | 0.139 |
| **48** | 0.726 | 0.748 | 3.096 | 1.042 | 0.041 | 3.941 | 0.089 |
| **49** | 0.243 | 0.229 | 3.084 | 0.816 | 0.021 | 3.930 | 0.057 |
| **TFR** | **2.180** | **2.183** | **2.183** | **2.184** | **2.148** | **2.183** | **2.143** |
| **SSE** |  | | 0.000456 | 0.000174 | 0.000565 | 0.00039 | 0.002009 |
| **K** |  | | 5 | 4 | 5 | 4 | 3 |
| **AICc** |  | | -379.581 | **-414.8** | -372.076 | -386.498 | -331.886 |
| $\boldsymbol{R}^{\boldsymbol{2}}$ |  | | 0.9971 | **0.9989** | 0.9968 | 0.9975 | 0.9877 |

**Table A2: Observed, LOWESS smoothed and estimated values of ASFR (per 1000) for Punjab along with the values of SSE and AICc under different proposed models**

| **Age** | **observed ASFR (per 1000)** | **LOWESS Smoothed ASFR (per 1000)** | **Estimated ASFR (per 1000) (Punjab)** | | | | |
| --- | --- | --- | --- | --- | --- | --- | --- |
|  |  |  | **Modified P-K Model** | **Modified Gompertz Model** | **Modified Skew Normal Model** | **Modified G-P Model** | **Hadwiger Model** |
| **15** | 0.000 | 0.000 | 2.823 | 0.626 | 2.494 | 1.101 | 3.471 |
| **16** | 1.026 | 1.488 | 6.682 | 3.225 | 6.421 | 2.517 | 9.023 |
| **17** | 12.359 | 10.849 | 14.747 | 10.996 | 14.587 | 11.565 | 19.378 |
| **18** | 26.255 | 28.110 | 29.290 | 27.168 | 29.254 | 30.516 | 35.499 |
| **19** | 54.750 | 54.336 | 51.711 | 52.187 | 51.785 | 56.529 | 56.898 |
| **20** | 85.889 | 86.215 | 80.789 | 82.346 | 80.913 | 84.696 | 81.424 |
| **21** | 113.073 | 110.140 | 111.502 | 111.425 | 111.593 | 110.285 | 105.755 |
| **22** | 125.483 | 131.499 | 135.855 | 133.713 | 135.849 | 129.874 | 126.344 |
| **23** | 150.224 | 141.290 | 146.086 | 146.089 | 145.976 | 141.693 | 140.379 |
| **24** | 136.624 | 145.825 | 144.381 | 148.329 | 144.259 | 145.459 | 146.400 |
| **25** | 149.517 | 143.661 | 138.608 | 142.219 | 138.509 | 141.995 | 144.417 |
| **26** | 134.774 | 135.258 | 129.296 | 130.392 | 129.242 | 132.787 | 135.633 |
| **27** | 113.985 | 115.658 | 117.195 | 115.441 | 117.198 | 119.594 | 121.951 |
| **28** | 102.478 | 104.342 | 103.223 | 99.450 | 103.284 | 104.149 | 105.473 |
| **29** | 93.959 | 86.068 | 88.349 | 83.864 | 88.458 | 87.960 | 88.107 |
| **30** | 58.037 | 69.913 | 73.488 | 69.551 | 73.626 | 72.207 | 71.339 |
| **31** | 66.336 | 54.564 | 59.411 | 56.934 | 59.555 | 57.721 | 56.159 |
| **32** | 36.922 | 46.374 | 46.692 | 46.133 | 46.817 | 44.998 | 43.099 |
| **33** | 40.067 | 33.119 | 35.681 | 37.085 | 35.767 | 34.259 | 32.321 |
| **34** | 23.842 | 29.176 | 26.524 | 29.627 | 26.555 | 25.510 | 23.734 |
| **35** | 26.153 | 22.092 | 19.193 | 23.554 | 19.160 | 18.612 | 17.098 |
| **36** | 15.937 | 18.885 | 13.534 | 18.655 | 13.436 | 13.341 | 12.103 |
| **37** | 14.642 | 11.969 | 9.318 | 14.732 | 9.156 | 9.433 | 8.431 |
| **38** | 5.603 | 7.815 | 6.281 | 11.606 | 6.064 | 6.620 | 5.787 |
| **39** | 6.709 | 6.380 | 4.167 | 9.127 | 3.903 | 4.655 | 3.918 |
| **40** | 7.252 | 5.730 | 2.743 | 7.168 | 2.441 | 3.323 | 2.620 |
| **41** | 0.771 | 2.208 | 1.814 | 5.623 | 1.484 | 2.447 | 1.731 |
| **42** | 0.474 | 0.211 | 1.228 | 4.407 | 0.877 | 1.890 | 1.132 |
| **43** | 0.987 | 0.718 | 0.869 | 3.451 | 0.503 | 1.548 | 0.733 |
| **44** | 0.329 | 0.445 | 0.656 | 2.702 | 0.281 | 1.344 | 0.470 |
| **45** | 0.000 | 0.026 | 0.534 | 2.114 | 0.152 | 1.228 | 0.299 |
| **46** | 0.000 | 0.000 | 0.466 | 1.654 | 0.080 | 1.165 | 0.188 |
| **47** | 0.000 | 0.000 | 0.429 | 1.293 | 0.041 | 1.131 | 0.118 |
| **48** | 0.000 | 0.000 | 0.410 | 1.011 | 0.021 | 1.115 | 0.073 |
| **49** | 0.000 | 0.011 | 0.400 | 0.791 | 0.010 | 1.107 | 0.045 |
| **TFR** | **1.604** | **1.604** | **1.604** | **1.635** | **1.600** | **1.604** | **1.602** |
| **SSE** |  | | 0.000316 | 0.000222 | 0.000318 | 0.000149 | 0.002009 |
| **K** |  | | 5 | 4 | 5 | 4 | 3 |
| **AICc** |  | | -389.052 | -405.284 | -388.852 | **-419.288** | -382.84 |
| $\boldsymbol{R}^{\boldsymbol{2}}$ |  | | 0.9966 | 0.9981 | 0.9966 | **0.9984** | 0.9949 |

**Table A3: Observed, LOWESS smoothed and estimated values of ASFR (per 1000) for Uttar Pradesh along with the values of SSE and AICc under different proposed models**

| **Age** | **Observed ASFR (per 1000)** | **LOWESS Smoothed ASFR (per 1000)** | **Estimated ASFR (per 1000) (Uttar Pradesh)** | | | | |
| --- | --- | --- | --- | --- | --- | --- | --- |
|  |  |  | **Modified P-K Model** | **Modified Gompertz Model** | **Modified Skew Normal Model** | **Modified G-P Model** | **Hadwiger Model** |
| **15** | 0.598 | 1.499 | 5.453 | 2.295 | 2.444 | 5.448 | 8.564 |
| **16** | 3.255 | 1.106 | 10.000 | 8.769 | 7.536 | 8.922 | 19.628 |
| **17** | 14.871 | 15.321 | 21.308 | 24.282 | 19.789 | 26.231 | 38.150 |
| **18** | 40.162 | 44.047 | 44.548 | 52.058 | 44.262 | 57.914 | 64.654 |
| **19** | 93.159 | 89.518 | 83.539 | 91.065 | 84.326 | 98.131 | 97.656 |
| **20** | 137.660 | 139.878 | 135.723 | 135.517 | 136.841 | 139.670 | 133.779 |
| **21** | 184.540 | 183.230 | 188.648 | 177.340 | 189.145 | 176.412 | 168.581 |
| **22** | 215.992 | 215.953 | 223.293 | 209.526 | 222.687 | 204.273 | 197.687 |
| **23** | 231.142 | 230.621 | 228.121 | 228.237 | 226.990 | 221.291 | 217.788 |
| **24** | 229.621 | 229.014 | 223.584 | 233.071 | 222.532 | 227.293 | 227.204 |
| **25** | 217.793 | 220.518 | 214.607 | 226.095 | 213.801 | 223.388 | 225.951 |
| **26** | 209.867 | 206.417 | 201.741 | 210.557 | 201.306 | 211.455 | 215.412 |
| **27** | 184.812 | 187.392 | 185.746 | 189.826 | 185.753 | 193.706 | 197.813 |
| **28** | 166.208 | 165.460 | 167.521 | 166.778 | 167.975 | 172.347 | 175.691 |
| **29** | 144.276 | 143.424 | 148.014 | 143.555 | 148.863 | 149.357 | 151.453 |
| **30** | 118.908 | 120.186 | 128.148 | 121.567 | 129.288 | 126.357 | 127.107 |
| **31** | 101.006 | 100.186 | 108.751 | 101.622 | 110.043 | 104.564 | 104.128 |
| **32** | 84.953 | 86.555 | 90.501 | 84.079 | 91.791 | 84.798 | 83.461 |
| **33** | 75.734 | 72.764 | 73.899 | 68.996 | 75.035 | 67.521 | 65.584 |
| **34** | 57.617 | 60.899 | 59.262 | 56.252 | 60.112 | 52.909 | 50.616 |
| **35** | 53.274 | 51.230 | 46.733 | 45.625 | 47.195 | 40.918 | 38.427 |
| **36** | 42.945 | 43.256 | 36.307 | 36.854 | 36.312 | 31.353 | 28.738 |
| **37** | 33.302 | 33.658 | 27.863 | 29.671 | 27.381 | 23.928 | 21.198 |
| **38** | 26.988 | 27.140 | 21.202 | 23.827 | 20.233 | 18.317 | 15.440 |
| **39** | 22.035 | 21.118 | 16.081 | 19.094 | 14.653 | 14.188 | 11.115 |
| **40** | 14.502 | 15.699 | 12.242 | 15.276 | 10.399 | 11.231 | 7.916 |
| **41** | 12.299 | 11.295 | 9.433 | 12.206 | 7.233 | 9.170 | 5.582 |
| **42** | 7.855 | 8.517 | 7.428 | 9.742 | 4.930 | 7.775 | 3.900 |
| **43** | 6.581 | 6.500 | 6.030 | 7.770 | 3.293 | 6.858 | 2.702 |
| **44** | 5.332 | 4.500 | 5.079 | 6.192 | 2.156 | 6.274 | 1.857 |
| **45** | 2.016 | 3.565 | 4.446 | 4.933 | 1.383 | 5.915 | 1.267 |
| **46** | 5.297 | 4.097 | 4.035 | 3.928 | 0.870 | 5.701 | 0.858 |
| **47** | 3.804 | 3.630 | 3.774 | 3.126 | 0.536 | 5.580 | 0.578 |
| **48** | 1.686 | 2.193 | 3.612 | 2.488 | 0.324 | 5.513 | 0.386 |
| **49** | 0.000 | 0.000 | 3.514 | 1.979 | 0.192 | 5.478 | 0.257 |
| **TFR** | **2.750** | **2.750** | **2.750** | **2.754** | **2.718** | **2.750** | **2.711** |
| **SSE** |  | | 0.000739 | 0.000542 | 0.000852 | 0.001593 | 0.003708 |
| **K** |  | | 5 | 4 | 5 | 4 | 3 |
| **AICc** |  | | -357.261 | **-372.752** | -352.283 | -335.004 | -310.43 |
| $\boldsymbol{R}^{\boldsymbol{2}}$ |  | | 0.9967 | **0.9976** | 0.9964 | 0.9928 | 0.9837 |

**Table A4: Observed, LOWESS smoothed and estimated values of ASFR (per 1000) for West Bengal along with the values of SSE and AICc under different proposed models**

| **Age** | **Observed ASFR (per 1000)** | **LOWESS Smoothed ASFR (per 1000)** | **Estimated ASFR (per 1000) (West Bengal)** | | | | |
| --- | --- | --- | --- | --- | --- | --- | --- |
|  |  |  | **Modified P-K Model** | **Modified Gompertz Model** | **Modified Skew Normal Model** | **Modified G-P Model** | **Hadwiger Model** |
| **15** | 13.721 | 7.233 | 16.105 | 29.307 | 15.593 | 1.406 | 40.831 |
| **16** | 38.382 | 50.847 | 44.554 | 57.434 | 44.538 | 52.768 | 65.565 |
| **17** | 88.126 | 93.564 | 92.938 | 91.606 | 93.237 | 101.129 | 93.175 |
| **18** | 141.879 | 138.752 | 142.941 | 124.498 | 143.056 | 134.635 | 119.632 |
| **19** | 163.943 | 162.270 | 161.539 | 149.404 | 161.220 | 153.669 | 141.054 |
| **20** | 159.046 | 160.691 | 159.455 | 162.765 | 159.138 | 160.752 | 154.726 |
| **21** | 153.540 | 155.057 | 154.098 | 164.478 | 153.838 | 158.784 | 159.566 |
| **22** | 152.512 | 150.955 | 145.801 | 156.776 | 145.640 | 150.459 | 156.043 |
| **23** | 141.434 | 143.504 | 135.064 | 142.798 | 135.030 | 138.056 | 145.729 |
| **24** | 125.903 | 118.138 | 122.505 | 125.554 | 122.606 | 123.401 | 130.736 |
| **25** | 84.747 | 96.849 | 108.799 | 107.404 | 109.024 | 107.880 | 113.224 |
| **26** | 97.193 | 90.370 | 94.622 | 89.939 | 94.944 | 92.500 | 95.057 |
| **27** | 85.995 | 84.749 | 80.594 | 74.075 | 80.973 | 77.946 | 77.636 |
| **28** | 62.124 | 65.620 | 67.242 | 60.228 | 67.631 | 64.648 | 61.872 |
| **29** | 52.660 | 50.063 | 54.968 | 48.482 | 55.320 | 52.837 | 48.240 |
| **30** | 39.488 | 42.271 | 44.043 | 38.723 | 44.315 | 42.596 | 36.880 |
| **31** | 38.066 | 34.717 | 34.607 | 30.742 | 34.765 | 33.904 | 27.700 |
| **32** | 27.863 | 32.031 | 26.689 | 24.292 | 26.710 | 26.668 | 20.475 |
| **33** | 31.648 | 27.152 | 20.225 | 19.125 | 20.097 | 20.752 | 14.917 |
| **34** | 18.299 | 21.334 | 15.087 | 15.014 | 14.809 | 16.000 | 10.725 |
| **35** | 14.827 | 13.402 | 11.108 | 11.761 | 10.687 | 12.246 | 7.619 |
| **36** | 9.001 | 9.883 | 8.104 | 9.197 | 7.553 | 9.330 | 5.354 |
| **37** | 7.012 | 6.446 | 5.892 | 7.182 | 5.227 | 7.103 | 3.724 |
| **38** | 3.653 | 3.526 | 4.302 | 5.603 | 3.543 | 5.431 | 2.567 |
| **39** | 1.036 | 1.899 | 3.187 | 4.367 | 2.352 | 4.199 | 1.754 |
| **40** | 2.919 | 2.144 | 2.423 | 3.402 | 1.529 | 3.306 | 1.190 |
| **41** | 1.898 | 1.903 | 1.913 | 2.649 | 0.973 | 2.673 | 0.801 |
| **42** | 0.590 | 1.158 | 1.579 | 2.062 | 0.607 | 2.232 | 0.536 |
| **43** | 1.551 | 0.818 | 1.366 | 1.604 | 0.371 | 1.931 | 0.356 |
| **44** | 0.000 | 0.629 | 1.233 | 1.248 | 0.222 | 1.732 | 0.235 |
| **45** | 0.631 | 0.218 | 1.152 | 0.971 | 0.130 | 1.602 | 0.155 |
| **46** | 0.000 | 0.195 | 1.104 | 0.755 | 0.074 | 1.520 | 0.101 |
| **47** | 0.000 | 0.034 | 1.076 | 0.587 | 0.042 | 1.470 | 0.066 |
| **48** | 0.000 | 0.000 | 1.060 | 0.456 | 0.023 | 1.441 | 0.043 |
| **49** | 0.000 | 0.005 | 1.051 | 0.355 | 0.012 | 1.424 | 0.028 |
| **TFR** | **1.760** | **1.768** | **1.768** | **1.765** | **1.756** | **1.768** | **1.738** |
| **SSE** |  | | 0.000597 | 0.001555 | 0.00061 | 0.000564 | 0.003304 |
| **K** |  | | 5 | 4 | 5 | 4 | 3 |
| **AICc** |  | | **-366.825** | -337.177 | -366.061 | -372.685 | -314.467 |
| $\boldsymbol{R}^{\boldsymbol{2}}$ |  | | **0.9947** | 0.9861 | 0.9946 | 0.9950 | 0.9709 |

**Table A5: Observed, LOWESS smoothed and estimated values of ASFR (per 1000) for Tripura along with the values of SSE and AICc under different proposed models**

| **Age** | **Observed ASFR (per 1000)** | **LOWESS Smoothed ASFR (per 1000)** | **Estimated ASFR (per 1000) (Tripura)** | | | | |
| --- | --- | --- | --- | --- | --- | --- | --- |
|  |  |  | **Modified P-K Model** | **Modified Gompertz Model** | **Modified Skew Normal Model** | **Modified G-P Model** | **Hadwiger Model** |
| **15** | 28.595 | 27.492 | 26.315 | 39.917 | 26.244 | 2.232 | 47.294 |
| **16** | 55.456 | 54.895 | 52.591 | 62.079 | 52.620 | 55.705 | 66.729 |
| **17** | 73.007 | 83.580 | 86.759 | 85.377 | 86.822 | 91.761 | 86.262 |
| **18** | 120.265 | 114.387 | 117.876 | 106.272 | 117.886 | 114.560 | 103.704 |
| **19** | 138.771 | 142.567 | 131.800 | 122.002 | 131.719 | 127.169 | 117.308 |
| **20** | 146.061 | 135.359 | 130.984 | 131.166 | 130.897 | 132.069 | 126.032 |
| **21** | 110.581 | 127.935 | 128.095 | 133.710 | 128.018 | 131.273 | 129.578 |
| **22** | 139.141 | 126.154 | 123.329 | 130.544 | 123.272 | 126.408 | 128.279 |
| **23** | 114.182 | 113.782 | 116.900 | 123.068 | 116.870 | 118.772 | 122.902 |
| **24** | 86.060 | 99.076 | 109.091 | 112.772 | 109.093 | 109.395 | 114.438 |
| **25** | 110.297 | 92.263 | 100.228 | 100.983 | 100.262 | 99.075 | 103.926 |
| **26** | 74.121 | 92.848 | 90.661 | 88.753 | 90.725 | 88.420 | 92.325 |
| **27** | 97.926 | 80.556 | 80.740 | 76.831 | 80.829 | 77.884 | 80.435 |
| **28** | 59.976 | 71.952 | 70.797 | 65.697 | 70.902 | 67.788 | 68.874 |
| **29** | 63.109 | 57.934 | 61.124 | 55.620 | 61.235 | 58.354 | 58.069 |
| **30** | 55.324 | 58.025 | 51.963 | 46.709 | 52.070 | 49.719 | 48.286 |
| **31** | 51.525 | 48.176 | 43.500 | 38.969 | 43.594 | 41.955 | 39.654 |
| **32** | 31.699 | 32.696 | 35.864 | 32.339 | 35.935 | 35.087 | 32.201 |
| **33** | 19.132 | 21.677 | 29.123 | 26.722 | 29.165 | 29.098 | 25.884 |
| **34** | 24.224 | 22.393 | 23.298 | 22.003 | 23.305 | 23.950 | 20.616 |
| **35** | 24.473 | 24.531 | 18.366 | 18.066 | 18.335 | 19.583 | 16.282 |
| **36** | 21.045 | 20.576 | 14.273 | 14.799 | 14.203 | 15.926 | 12.761 |
| **37** | 14.355 | 15.311 | 10.940 | 12.101 | 10.832 | 12.905 | 9.932 |
| **38** | 10.874 | 9.880 | 8.278 | 9.879 | 8.134 | 10.441 | 7.680 |
| **39** | 4.715 | 5.552 | 6.190 | 8.056 | 6.014 | 8.459 | 5.904 |
| **40** | 2.846 | 2.579 | 4.583 | 6.562 | 4.377 | 6.886 | 4.514 |
| **41** | 1.411 | 1.272 | 3.368 | 5.341 | 3.137 | 5.657 | 3.434 |
| **42** | 0.000 | 0.125 | 2.465 | 4.345 | 2.214 | 4.710 | 2.600 |
| **43** | 0.000 | 0.259 | 1.807 | 3.532 | 1.538 | 3.993 | 1.960 |
| **44** | 1.190 | 0.638 | 1.334 | 2.870 | 1.052 | 3.458 | 1.472 |
| **45** | 0.000 | 0.259 | 1.001 | 2.332 | 0.709 | 3.068 | 1.102 |
| **46** | 0.000 | 0.346 | 0.770 | 1.894 | 0.470 | 2.789 | 0.821 |
| **47** | 1.417 | 0.518 | 0.613 | 1.537 | 0.307 | 2.593 | 0.610 |
| **48** | 0.000 | 0.381 | 0.508 | 1.248 | 0.197 | 2.459 | 0.452 |
| **49** | 0.000 | 0.000 | 0.439 | 1.013 | 0.125 | 2.371 | 0.334 |
| **TFR** | **1.682** | **1.686** | **1.686** | **1.695** | **1.683** | **1.686** | **1.683** |
| **SSE** |  | | 0.000604 | 0.001599 | 0.000605 | 0.001527 | 0.002221 |
| **K** |  | | 5 | 4 | 5 | 4 | 3 |
| **AICc** |  | | **-366.374** | -336.202 | -366.321 | -337.806 | -328.363 |
| $\boldsymbol{R}^{\boldsymbol{2}}$ |  | | **0.9922** | 0.9795 | 0.9922 | 0.9803 | 0.9714 |

**Table A6: Observed, LOWESS smoothed and estimated values of ASFR (per 1000) for Gujarat along with the values of SSE and AICc under different proposed models**

| **Age** | **Observed ASFR (per 1000)** | **LOWESS Smoothed ASFR (per 1000)** | **Estimated ASFR (per 1000) (Gujarat)** | | | | |
| --- | --- | --- | --- | --- | --- | --- | --- |
|  |  |  | **Modified P-K Model** | **Modified Gompertz Model** | **Modified Skew Normal Model** | **Modified G-P Model** | **Hadwiger Model** |
| **15** | 1.384 | 0.472 | 6.539 | 3.473 | 3.437 | 2.636 | 10.124 |
| **16** | 9.737 | 11.322 | 14.462 | 12.425 | 11.688 | 8.415 | 22.580 |
| **17** | 30.226 | 31.619 | 30.974 | 31.794 | 29.891 | 31.120 | 42.468 |
| **18** | 63.013 | 61.821 | 59.395 | 62.645 | 60.396 | 66.191 | 69.315 |
| **19** | 98.133 | 99.784 | 99.046 | 100.668 | 100.773 | 104.729 | 100.426 |
| **20** | 141.041 | 140.974 | 142.145 | 137.988 | 143.061 | 139.157 | 131.516 |
| **21** | 175.845 | 176.088 | 174.887 | 167.081 | 175.302 | 164.731 | 157.972 |
| **22** | 187.515 | 178.219 | 184.534 | 183.652 | 179.234 | 179.472 | 176.132 |
| **23** | 157.885 | 174.226 | 181.241 | 187.195 | 179.464 | 183.520 | 184.090 |
| **24** | 188.982 | 177.081 | 173.265 | 179.899 | 173.634 | 178.376 | 181.853 |
| **25** | 171.556 | 175.013 | 161.234 | 165.129 | 162.709 | 166.220 | 170.960 |
| **26** | 150.231 | 148.877 | 146.059 | 146.240 | 147.928 | 149.402 | 153.844 |
| **27** | 124.938 | 127.591 | 128.817 | 125.944 | 130.631 | 130.101 | 133.175 |
| **28** | 113.120 | 113.793 | 110.629 | 106.127 | 112.127 | 110.128 | 111.367 |
| **29** | 97.912 | 90.539 | 92.539 | 87.921 | 93.589 | 90.854 | 90.296 |
| **30** | 53.738 | 61.990 | 75.424 | 71.879 | 75.976 | 73.208 | 71.208 |
| **31** | 50.345 | 49.070 | 59.935 | 58.160 | 59.996 | 57.727 | 54.767 |
| **32** | 50.790 | 46.486 | 46.476 | 46.681 | 46.087 | 44.633 | 41.180 |
| **33** | 34.613 | 39.738 | 35.216 | 37.233 | 34.440 | 33.912 | 30.335 |
| **34** | 37.002 | 33.588 | 26.130 | 29.552 | 25.036 | 25.393 | 21.933 |
| **35** | 26.882 | 27.510 | 19.048 | 23.366 | 17.705 | 18.812 | 15.591 |
| **36** | 17.319 | 18.249 | 13.709 | 18.419 | 12.180 | 13.865 | 10.911 |
| **37** | 13.261 | 12.184 | 9.814 | 14.486 | 8.152 | 10.243 | 7.528 |
| **38** | 7.627 | 8.848 | 7.061 | 11.372 | 5.307 | 7.661 | 5.126 |
| **39** | 7.263 | 5.995 | 5.176 | 8.915 | 3.361 | 5.870 | 3.448 |
| **40** | 3.222 | 3.990 | 3.924 | 6.981 | 2.071 | 4.661 | 2.294 |
| **41** | 2.949 | 3.097 | 3.117 | 5.462 | 1.241 | 3.868 | 1.511 |
| **42** | 3.963 | 3.335 | 2.613 | 4.271 | 0.724 | 3.362 | 0.985 |
| **43** | 1.871 | 2.123 | 2.307 | 3.337 | 0.411 | 3.051 | 0.637 |
| **44** | 0.396 | 0.384 | 2.126 | 2.607 | 0.227 | 2.864 | 0.408 |
| **45** | 0.000 | 0.280 | 2.023 | 2.036 | 0.122 | 2.757 | 0.260 |
| **46** | 1.299 | 0.979 | 1.966 | 1.589 | 0.064 | 2.698 | 0.164 |
| **47** | 1.295 | 1.290 | 1.935 | 1.241 | 0.032 | 2.666 | 0.103 |
| **48** | 1.119 | 0.967 | 1.919 | 0.968 | 0.016 | 2.650 | 0.064 |
| **49** | 0.000 | 0.071 | 1.911 | 0.755 | 0.008 | 2.642 | 0.040 |
| **TFR** | **2.026** | **2.028** | **2.028** | **2.047** | **1.997** | **2.028** | **2.005** |
| **SSE** |  | | 0.000862 | 0.000747 | 0.000839 | 0.00081 | 0.001638 |
| **K** |  | | 5 | 4 | 5 | 4 | 3 |
| **AICc** |  | | -351.878 | **-361.523** | -352.813 | -358.665 | -339.021 |
| $\boldsymbol{R}^{\boldsymbol{2}}$ |  | | 0.9940 | **0.9950** | 0.9945 | 0.9944 | 0.9889 |

**Table A7: Observed, LOWESS smoothed and estimated values of ASFR (per 1000) for Karnataka along with the values of SSE and AICc under different proposed models**

| **Age** | **Observed ASFR (per 1000)** | **LOWESS Smoothed ASFR (per 1000)** | **Estimated ASFR (per 1000) (Karnataka)** | | | | |
| --- | --- | --- | --- | --- | --- | --- | --- |
|  |  |  | **Modified P-K Model** | **Modified Gompertz Model** | **Modified Skew Normal Model** | **Modified G-P Model** | **Hadwiger Model** |
| **15** | 5.881 | 5.551 | 5.141 | 3.885 | 3.341 | 2.034 | 11.832 |
| **16** | 11.447 | 10.885 | 13.662 | 14.811 | 12.416 | 10.026 | 26.442 |
| **17** | 28.651 | 32.462 | 33.815 | 38.450 | 33.592 | 38.518 | 49.309 |
| **18** | 62.943 | 71.424 | 70.247 | 74.500 | 70.754 | 78.733 | 79.100 |
| **19** | 133.503 | 121.186 | 118.782 | 115.525 | 119.436 | 118.881 | 111.810 |
| **20** | 149.853 | 159.886 | 162.017 | 151.191 | 162.775 | 150.696 | 141.964 |
| **21** | 180.766 | 169.814 | 177.862 | 173.892 | 174.354 | 170.258 | 164.440 |
| **22** | 165.299 | 177.960 | 174.834 | 181.266 | 173.831 | 177.095 | 175.985 |
| **23** | 184.115 | 172.834 | 166.801 | 175.317 | 167.188 | 172.912 | 175.843 |
| **24** | 154.182 | 160.570 | 154.461 | 160.163 | 155.485 | 160.462 | 165.478 |
| **25** | 138.320 | 135.723 | 138.840 | 140.100 | 140.040 | 142.729 | 147.738 |
| **26** | 116.752 | 119.338 | 121.154 | 118.564 | 122.269 | 122.414 | 125.909 |
| **27** | 103.284 | 99.478 | 102.649 | 97.843 | 103.541 | 101.678 | 102.972 |
| **28** | 78.283 | 83.083 | 84.465 | 79.210 | 85.067 | 82.064 | 81.176 |
| **29** | 71.326 | 66.581 | 67.528 | 63.195 | 67.815 | 64.533 | 61.924 |
| **30** | 49.930 | 53.761 | 52.484 | 49.861 | 52.459 | 49.562 | 45.864 |
| **31** | 42.963 | 40.814 | 39.695 | 39.009 | 39.378 | 37.265 | 33.078 |
| **32** | 29.768 | 29.504 | 29.260 | 30.322 | 28.684 | 27.506 | 23.289 |
| **33** | 17.478 | 19.748 | 21.070 | 23.455 | 20.276 | 20.000 | 16.043 |
| **34** | 16.643 | 13.324 | 14.880 | 18.075 | 13.908 | 14.397 | 10.834 |
| **35** | 8.469 | 12.986 | 10.369 | 13.889 | 9.258 | 10.332 | 7.185 |
| **36** | 17.077 | 12.571 | 7.195 | 10.650 | 5.980 | 7.463 | 4.687 |
| **37** | 8.182 | 10.525 | 5.039 | 8.153 | 3.748 | 5.494 | 3.011 |
| **38** | 5.752 | 5.330 | 3.623 | 6.233 | 2.280 | 4.180 | 1.908 |
| **39** | 3.454 | 2.986 | 2.725 | 4.761 | 1.346 | 3.327 | 1.193 |
| **40** | 0.404 | 1.367 | 2.173 | 3.634 | 0.771 | 2.790 | 0.737 |
| **41** | 1.759 | 0.712 | 1.845 | 2.772 | 0.428 | 2.463 | 0.451 |
| **42** | 0.223 | 1.224 | 1.657 | 2.114 | 0.231 | 2.269 | 0.273 |
| **43** | 2.037 | 1.154 | 1.552 | 1.611 | 0.121 | 2.158 | 0.164 |
| **44** | 0.498 | 0.978 | 1.495 | 1.228 | 0.061 | 2.097 | 0.097 |
| **45** | 0.316 | 0.113 | 1.465 | 0.936 | 0.030 | 2.064 | 0.057 |
| **46** | 0.000 | 0.270 | 1.451 | 0.713 | 0.015 | 2.048 | 0.034 |
| **47** | 0.680 | 0.272 | 1.443 | 0.543 | 0.007 | 2.040 | 0.019 |
| **48** | 0.000 | 0.174 | 1.440 | 0.414 | 0.003 | 2.036 | 0.011 |
| **49** | 0.000 | 0.000 | 1.438 | 0.315 | 0.001 | 2.035 | 0.006 |
| **TFR** | **1.790** | **1.795** | **1.795** | **1.807** | **1.771** | **1.795** | **1.771** |
| **SSE** |  | | 0.000282 | 0.000335 | 0.000289 | 0.000384 | 0.001676 |
| **K** |  | | 5 | 4 | 5 | 4 | 3 |
| **AICc** |  | | **-390.939** | -389.567 | -390.146 | -384.788 | -338.232 |
| $\boldsymbol{R}^{\boldsymbol{2}}$ |  | | **0.9978** | 0.9975 | 0.9980 | 0.9971 | 0.9874 |

**Appendix: B**

**Figure B1: Observed and expected Age-Specific Fertility Rate (ASFR) for India under P-K model and Modified P-K model**

**Figure B2: Observed and expected Age-Specific Fertility Rate (ASFR) for India under Gompertz model and Modified Gompertz model**

**Figure B3: Observed and expected Age-Specific Fertility Rate (ASFR) for India under Skew Normal model and Modified Skew Normal model**

**Figure B4: Observed and expected Age-Specific Fertility Rate (ASFR) for India under G-P model and Modified G-P model**
